# Analytical modelling of food storage cooling with solar ammonia-water absorption system, powered by parabolic trough collectors. Method

**DOI:** 10.1016/j.mex.2023.102013

**Published:** 2023-01-10

**Authors:** Octavian G. Pop, Alexandru Dobrovicescu, Alexandru Serban, Mihaela Ciocan, Anass Zaaoumi, Daniel P. Hiris, Mugur C. Balan

**Affiliations:** a Technical University of Cluj-Napoca, Bd. 21 Decembrie 1989, Cluj-Napoca 400641, Romania; b University Politehnica of Bucharest, Splaiul Independenţei nr. 313, Sector 6, Bucharest 060042, Romania; c Mohammed V University, Thermal and Energy Research Team, Energy Research Centre, ENSET, Rabat, Morocco; d Technical University of Cluj-Napoca, Bd. Muncii 103-105, Cluj-Napoca 400641, Romania

**Keywords:** Solar cooling, Absorption, Ammonia-water, Parabolic trough collectors, Food storage cooling, Modelling, Analytical modelling of food storage cooling with solar ammonia-water absorption system, powered by parabolic trough collectors

## Abstract

The study presents a new analytical model capable to reveal the thermal behaviour of all the components of the solar ammonia-water absorption system, powered by parabolic trough collectors, serving different types of food storages: refrigeration chamber, refrigerated food storage, freezing chamber and frozen food storage. The heat inputs, that determine the total cooling load, for each food storage spaces consist of: heat gains through walls, heat gains through ventilation (fresh air), heat that must be dissipated from the stored products (technological cooling load required to cool down the products) and heat gains through operation. The influence of the number of solar parabolic trough collectors and of the storage tank size on different parameters of the refrigeration plant are investigated under low and high storage temperatures.

• Food cooling with solar absorption refrigeration system.
• Hourly based variation of NH_3_-H_2_O solar absorption system performances.
• Long term simulation of solar absorption cooling for refrigeration and cooling.

## Graphical abstract

**Figure:**
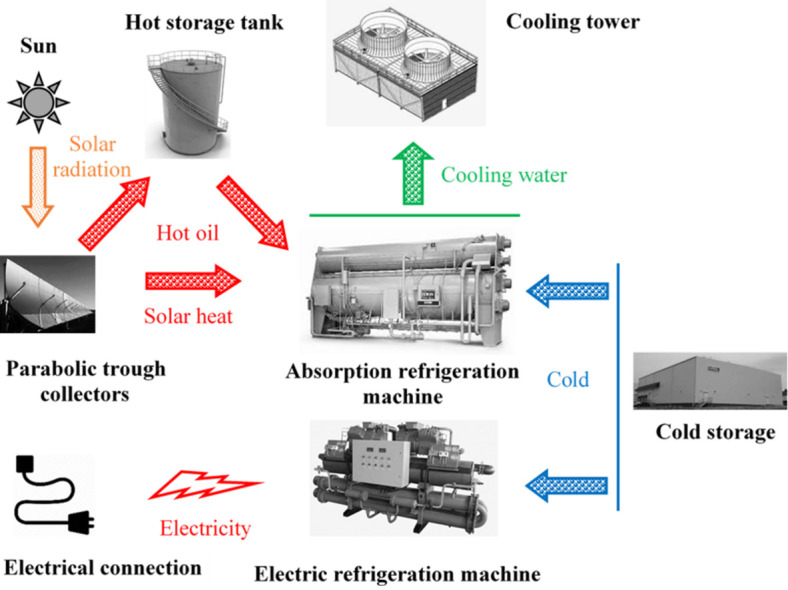

Specifications Table

**Table:** 

Subject Area:	Refrigeration, Solar Energy, and Analytical Modelling
More specific subject area:	Analytical modelling of solar refrigeration system
Method name:	Analytical modelling of food storage cooling with solar ammonia-water absorption system, powered by parabolic trough collectors
Name and reference of original method:	Not applicable
Resource availability:	Not applicable

## Introduction

In industrial solar thermal cooling applications, if the required temperatures are near 0 °C or below, the absorption-based systems with ammonia-water (NH_3_—H_2_O) are the most appropriate options [9]. The temperature of the hot heat transfer fluid required for driving such equipment are recommended in the range of (120 … 180) °C [1] or of (140 … 200) °C [27]. If solar energy is used to power these systems, solar collectors with concentrators (SCC) are required. Experimental studies investigating solar cooling below 0 °C provided by linear SCC, are reported in [27,10]. The basic principles of modelling solar parabolic trough collectors (SPTC) are presented in [20].

Modelling the NH_3—_H_2_O absorption cycles starts with the calculation of thermal properties of this solution, as presented in [30, 14] and later in [26,28].

A mathematical model of a solar NH_3—_H_2_O absorption system is provided in [2] and was applied to milk cooling applications in [3]. Thermal performances of a NH_3—_H_2_O refrigeration system are investigated in [18] while optimizations of such systems are proposed in [4]. Solar absorption refrigeration systems were modelled in [13] where a 5-days simulation is presented. A complex NH_3—_H_2_O absorption system providing freezing, air conditioning and heating was proposed in [11].

Combinations of different SCC and different types of LiBr-H_2_O absorption chillers with single and double effect were investigated in [25]. Parabolic dish collectors are investigated in solar refrigeration and desalinization systems [19]. SPTC can be used to power both NH_3—_H_2_O heat pumps [7] and adsorption refrigeration systems [15].

Heat storage must be used to allow continuous operation of the absorption refrigeration system, due to the well-known variability of solar radiation. Both hot or cold storage tanks can be used [12,21].

The number of investigations related to the solar refrigeration systems based on SCC is mentioned to be very low in some studies. In [27], it is stated that studies on the testing of NH_3—_H_2_O absorption systems with SCC are scarce. In [5], it is reported that the number of publications related to absorption systems with SCC are “far to be comparable” to the number of studies investigating the classic low temperature solar collectors. The same study mentions that in 2012 the total number of operational absorption systems with SCC was of only 46. A review related to these systems mentions that in 2011 < 30 systems based on these SCC were operational worldwide and only 6 of them could provide negative temperatures [1].

Long term simulations of the dynamic thermal behaviour of SCC based absorption systems are also rare, like the one-year simulations of solar LiBr-H_2_O single effect and double effect absorption systems that are presented in [5] and [31], respectively. Also, one-year simulations of a solar NH_3—_H_2_O absorption system is analysed in [16].

According to the presented literature review, the following aspects were not investigated:
- Food cooling with solar absorption refrigeration system.
- Hourly based modelling of food cooling load variation.
- Hourly based variation of NH_3—_H_2_O solar absorption system performances.
- Long term simulation of solar absorption cooling for refrigeration and cooling.

The goal of the study is to present the methodology for the analytical investigation of the food storage cooling with solar ammonia-water absorption system, powered by SPTC. The refrigeration plant considered in this study, consists of one stage NH_3—_H_2_O absorption, powered by SPTC. The heat is evacuated through water in a closed circuit equipped with a cooling tower. The backup equipment is a classic one stage mechanical compression refrigeration plant with NH_3_ as refrigerant. A seasonal heat storage is also considered.

## Method

### Preliminary considerations

This study is focused on the solar cooling of four types of food storages:
- Refrigeration chamber (RC) (fresh products are cooled above 0 °C),
- Refrigerated food storage (RS) (previously refrigerated products are maintained at above 0 °C),
- Freezing chamber (FC) (fresh products are cooled below 0 °C),
- Frozen food storage (FS) (previously frozen products are maintained at below 0 °C).

For simplicity, for this solar cooling study, it was considered that all types of the studied food storage spaces are employed for chicken meat. The influence of other products can also be investigated based on the same method.

The climatic data considered for calculations were taken from the typical meteorological year (TMY), available on the European Union web site [17], that provides hourly based variations of several climatic parameters: dry bulb (or ambient) temperature (t_db_ [ °C]), relative humidity (φ [%]), global solar radiation on the horizontal plane (I [W/m^2^]), direct solar radiation (I_dir_ [W/m^2^]), diffuse solar radiation (I_dif_ [W/m^2^]), direct normal irradiance (DNI [W/m^2^]), etc.

The cooling load types, considered for each food storage space, are presented in Table 1.

**Table 1:** Cooling load types for each food storage space.

Storage type	Walls^1^	Ventilation^2^	Technological^3^	Operation^4^
RC	✓		✓	✓
RS	✓	✓		✓
FC	✓		✓	✓
FS	✓			✓

1 Heat gains through walls;.

2 Heat gains through ventilation (fresh air);.

3 Technological cooling load (required to cool down the products);.

4 Heat gains through operation.

For all the cold spaces, each wall, ceiling, and floor, were considered to be manufactured from sandwich panels filled with polyurethane. The width of these panels was calculated considering the ambient temperature and relative humidity of 40 °C and 40%, respectively.

The inside temperature was considered to be 3 °C for refrigeration and −20 °C for freezing. The inside relative humidity was considered to be 90% in all cases.

The air flow rate for ventilation was considered to be 3 storage volumes in 24 h (15,000 m^3^/h).

The technological cooling load (required to cool down the chicken meat), for both refrigeration and freezing was determined based on the specific enthalpy variation with temperature, as presented in Table 2.

**Table 2:** Chicken meat specific enthalpy variation with temperature.

Temperature [ °**C]**	−18	−15	−10	−5	−1	0	1	5	10	15	20	25
Specific enthalpy [kJ/kg]	4.6	12.97	30.13	57.34	186.2	232.3	235.6	248.2	264.5	280.4	296.7	312.6

The cooling charge duration was considered to be 10 h for refrigeration and 20 h for freezing.

The main refrigeration equipment is of one stage absorption type with ammonia-water as working couple.

The backup refrigeration equipment, to be used in periods without available solar heat is a one stage mechanical refrigeration machine, with ammonia as refrigerant. In order to increase the availability of solar heat, a heat storage tank was proposed, and its influence was investigated.

For the heat rejection, from both thermal (absorption) and electrical (mechanical compression) machines, a cooling tower with closed water circuit is used.

**Fig. 1:**
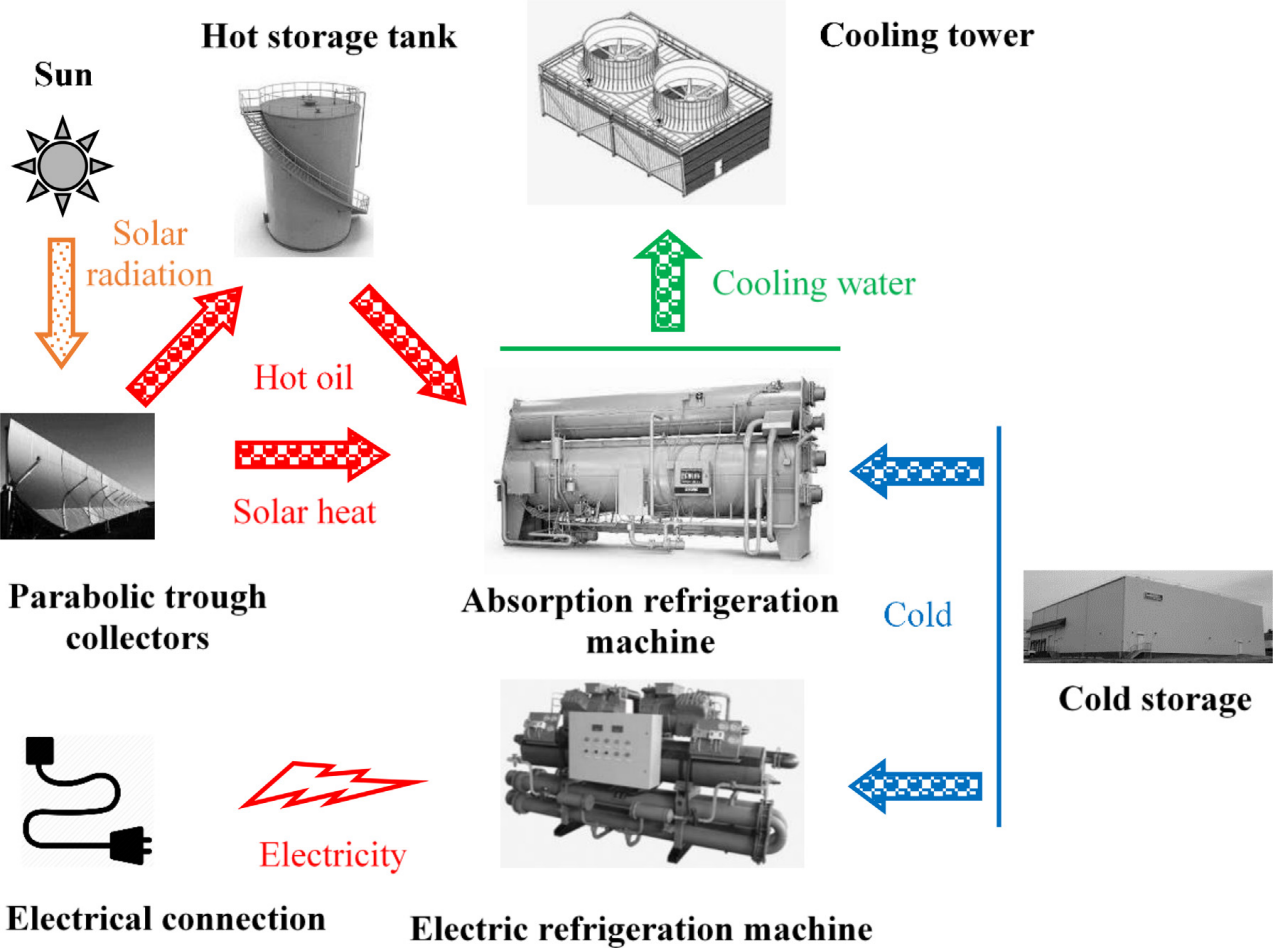
The principle scheme of the solar cooling system with electric backup.

The principle scheme of the investigated system is presented in Fig. 1.

It can be observed that cold can be extracted from the cold storage spaces by both the main absorption machine, driven by solar heat or by the mechanical machine, driven by electricity. The thermal behaviour and the energy exchange of both machines was investigated. In this study, the electric energy consumption of the pump was computed, but no particular attention was granted to the source of this electrical energy and to its value, that was neglected when the COP of the absorption machine was computed.

### Time variation of the cooling load through walls

The time variation of the cooling load (or heat gain) through walls is determined by the ambient temperature variation and the dependence between this type of cooling load and the ambient temperature is linear.

The cooling load through walls (${\dot{Q}}_{w}\left[W\right]$) can be determined with the Eq. (1):

(1) $${\dot{Q}}_{w}=k\cdot S\cdot \left(t_{db}-t_{i}\right)\left[W\right]$$

where:

k·S [W/K] is the thermal characteristic of the cold storage spaces
t_db_ [°C] is the variable dry bulb (ambient) temperature
t_i_ [°C] is the inside temperature of the cold storage spaces (3 °C for RC and RS and −20 °C for FC and FS)

The values of (k·S) for the investigated cold storage spaces are presented in the Table 3.

**Table 3:** The values of (k·S) for the food cold storage spaces.

Storage type	RC	RS	FC	FS
(k·S) [kW/K]	0.0135	0.676	0.0083	0.50

These values depend on each wall's level of insulation and on the dimensions of the storage.

The cooling load through walls (${\dot{Q}}_{w}\left[W\right]$) can also be determined with the Eq. (2):

(2) $${\dot{Q}}_{w}=\frac{{\dot{Q}}_{n}}{t_{n}-t_{i}}\cdot t_{db}-\frac{{\dot{Q}}_{n}\cdot t_{l}}{t_{n}-t_{i}}\left[W\right]$$

where:

${\dot{Q}}_{n}\left[W\right]$ is the nominal cooling load through walls, determined in nominal working conditions (0.5 kW for RC, 25 kW for RS, 0.5 kW for FC and 30 kW for FS)
t_n_ = 40 °C is the nominal dry bulb (ambient) temperature considered in this study

### Time variation of the ventilation cooling load

The single ventilated cold storage space is RS, and the ventilation cooling load is determined by the need to cool down the fresh air from the variable ambient temperature to the inside temperature.

The ventilation cooling load (${\dot{Q}}_{v}\left[W\right]$) can be calculated with the Eq. (3):

(3) $${\dot{Q}}_{v}=\rho \cdot \dot{V}\cdot c\cdot \left(t_{db}-t_{i}\right)\left[W\right]$$

where:

ρ [kg/m^3^] is the fresh air density
$\dot{V}$ [m^3^/s] is the fresh air volume flow rate calculated to supply the 3 vol air change in 24 h
c [kJ/kgK] is the fresh air specific heat capacity

### Time variation of the technological cooling load

The time variation of the technological cooling load was considered based on the shape of the cooling load curves presented in [6].

**Fig. 2:**
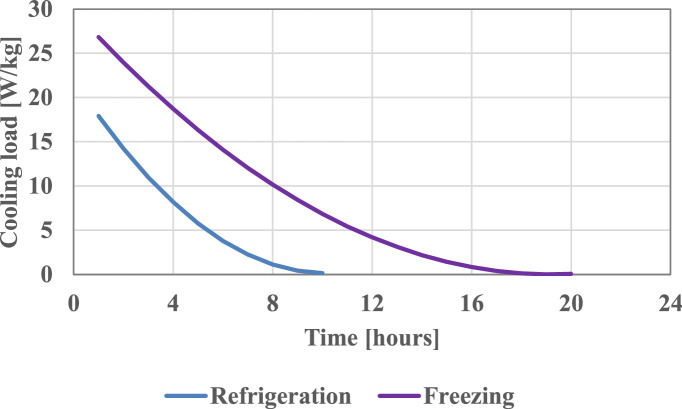
The variation of the technological cooling load for 1 kg of chicken meat.

Fig. 2 presents the considered cooling load variation in time, for both refrigeration and freezing of the chicken meat.

For the calculation of the technological cooling load (${\dot{Q}}_{t}\left[W/\text{kg}\right]$) in the case of chicken meat , the variation with time (τ [h]) was taken into account using the proposed equation:

(4) $${\dot{Q}}_{t}=a\cdot \tau^{2}-b\cdot \tau +c$$

The coefficients a, b and c, are provided in Table 4.

**Table 4:** Values of coefficients.

Coefficients	a	b	c
Refrigeration	0.2120	4.30615	21.9990
Freezing	0.0813	3.11665	29.8795

To the best of the authors' knowledge, such an equation is not available in the literature.

### Time variation of the operation cooling load

The operation cooling load is determined by the heat gain through the operations of loading, unloading, lighting, etc.

In this study, the operation cooling load (${\dot{Q}}_{op}\left[W\right]$) was determined as a share of the cooling load through walls with the Eq. (5):

(5) $${\dot{Q}}_{op}=0.4\cdot {\dot{Q}}_{w}$$

### Analytical model for the thermal efficiency of the parabolic trough collectors

The analytical model of the SPTC, is presented, and validated in [29]. Some information about SPTC is also presented in [20]. The location is determined by longitude (λ [°]) and latitude (φ [°]) while the position of the sun is defined by the angle of the solar altitude (γ_S_ [°]) and the angle of solar azimuth (α_S_ [°]). The orientation of the collectors is defined by the tilt angle (γ_t_ [°]) and the orientation angle related to the azimuth (α_t_ [°]). The solar angle of incidence on the tilted surface of the SPTC (θ [°]) and the global thermal efficiency of the SPTC (η [-]) were computed.

### The thermal regime of the cooling tower

The investigation of the thermal regime of the cooling tower during the whole year operation period is important because this equipment is used by both types of refrigerating plants: the solar driven absorption plant and the electrical driven plant used as backup.

The cooling water temperature (at the return from the cooling tower) was determined as a function of the wet bulb temperature (t_wb_ [ °C]):

(6) $$t_{w}=t_{wb}+5\left[{}^{\circ }C\right]$$

The difference of 5 °C is in agreement with [22] where this temperature difference, for such kind of applications, is reported in the range (3.2 – 4.8) °C and with [23] where this temperature difference is reported in the range (1.5 – 5.5) °C.

The wet bulb temperature is determined by the ambient (dry bulb) temperature (t_db_ [°C]) and by the relative humidity (φ [%]) of the ambient air and was calculated using the equation provided in [24]:

(7) $$\begin{array}{ccc} t_{wb} & = & t_{db}\cdot atan\left[0.151977\cdot {\left(\varphi +8.313659\right)}^{1/2}\right]+atan\left(t_{db}+\varphi \right)\\ & & -\;atan\left(\varphi -1.676331\right)+0.00391838\cdot \varphi^{3/2}\cdot atan\left(0.023101\cdot \varphi \right)-4.686035 \end{array}$$

The operation of the cooling tower was considered adjustable to maintain a minimum temperature of the cooling water at outlet of 10 °C. Thus, if the ambient temperature decreases, the fans speed will be reduced, to maintain the minimum set up temperature of the cooling water.

### Thermal behaviour of the storage tank

The storage tank is considered fully mixed, is filled with diathermic oil, and operates as a heat buffer to power the absorption plant in periods with reduced or without solar radiation. The tank is heated when solar radiation is available in excess and is cooled down when the stored heat is used.

The maximum temperature of the oil in the storage tank can be either the maximum expected value of the required hot temperature or the higher temperature that can be reached in the SPTC (400 °C). In this study both situations were considered. A heat exchanger is considered to provide the exact required variable heating power at the required variable temperature, according to the variable operating conditions of the plant. The heating power and temperature are regulated depending on the available oil temperature in the storage tank, by also regulating the oil flow rate.

The minimum temperature of the oil in the storage tank should be the minimum required hot temperature for the operation of the absorption plant, depending on the operating conditions.

The temperature variation of the storage tank oil (Δt [ °C]) in a period (τ [s]), can be determined as a function of the exchanged heat (Q_st_ [kJ]):

(8) $$\Delta t=\frac{Q_{st}}{m\cdot c}$$

where:

- m [kg] is the mass of the oil in the storage tank
- *c* = 1.85 kJ/kgK is the average specific heat capacity of the oil.

The exchanged heat can be calculated from the heat balance on the storage tank:

(9) $$Q_{st}=Q_{sol,ex}-Q_{op,st}-Q_{loss}$$

where:

- Q_sol,ex_ [kJ] is the solar heat that exceeds the required heat for the absorption plant operation
- Q_op,st_ [kJ] is the heat extracted from the storage tank for the absorption plant operation
- Q_loss_ [kJ] is the heat loss through the storage tank insulation under the variable temperature difference between the oil and the exterior air.

A layer of polyurethane thermal insulation with a thickness of 0.5 m was considered.

### Absorption refrigeration plant

**Fig. 3:**
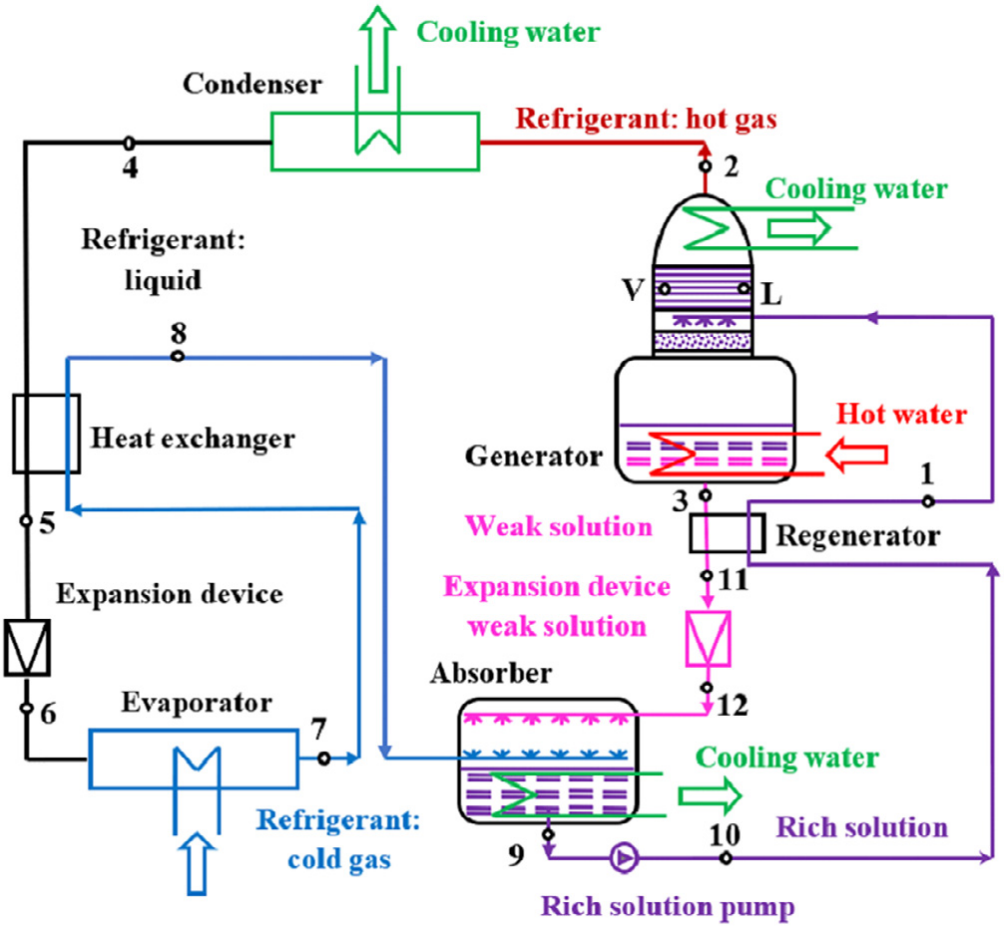
The principle scheme of the NH_3—_H_2_O absorption refrigerating plant.

The principle scheme of the main one stage absorption refrigerating plant with NH_3—_H_2_O is presented in Fig. 3.

**Fig. 4:**
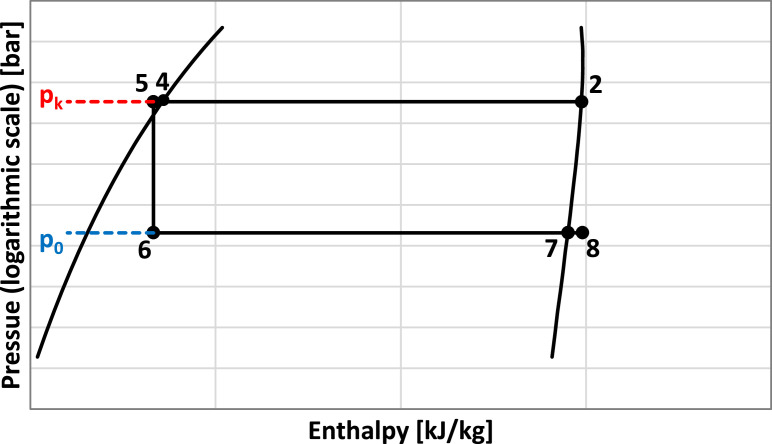
The working process of the main refrigerating circuit.

**Fig. 5:**
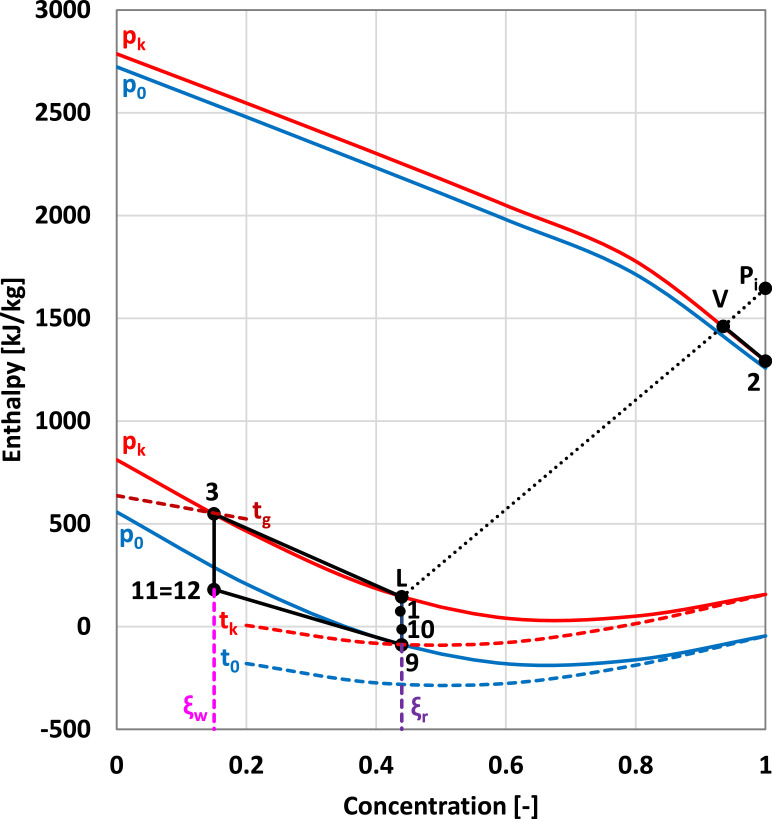
The working process of the NH_3—_H_2_O solution.

The main refrigerating circuit is composed of the condenser, the expansion device, the evaporator, and the heat exchanger, while the thermochemical compressor consists of the following components: absorber; rich solution pump; generator; expansion device of the weak solution and heat exchanger. The refrigerant is NH_3_ and the solvent is H_2_O. The working process of the main refrigerating circuit is presented in Fig. 4 in the pressure – enthalpy diagram, while the working process of the NH_3—_H_2_O solution is presented in Fig. 5 in the enthalpy – concentration diagram.

The internal working conditions were determined as a function of the external working conditions.

The evaporating temperature (t_0_ [ °C]) was determined as a function of the inside temperature (t_i_ [ °C]):

(10) $$t_{0}=t_{7}=t_{i}-13\left[{}^{\circ }C\right]$$

The assumption that the vapors of the refrigerant at the outlet of the evaporator are at the evaporating temperature agrees with [8]. The evaporating temperature determines the evaporating pressure (p_0_ [bar]).

The condensing temperature (t_k_ [ °C]) was determined as a function of the cooling water temperature (t_w_ [ °C]) at the return from the cooling tower:

(11) $$t_{k}=t_{4}=t_{w}+8\left[{}^{\circ }C\right]$$

The assumption that the liquid refrigerant at the outlet of the condenser is saturated, agrees with [4]. The condensing temperature determines the condensing pressure (p_k_ [bar]).

The saturated weak solution temperature at the outlet of the generator (t_g_ [ °C]) was determined as a function of the hot oil temperature (t_h_ [ °C]) at the return from the SPTC:

(12) $$t_{g}=t_{3}=t_{h}-10\left[{}^{\circ }C\right]$$

The difference of 10 °C agrees with [2].

The superheated refrigerant temperature (t_8_ [°C]) at the outlet of the heat exchanger from the main refrigerant circuit, was determined as:

(13) $$t_{8}=t_{k}-20\left[{}^{\circ }C\right]$$

The specific enthalpy of the subcooled refrigerant (h_5_ [kJ/kg]) at the outlet of the heat exchanger from the main refrigerant circuit, was determined from the energy balance equation on the heat exchanger:

(14) $$h_{5}=h_{4}-h_{8}+h_{7}\left[kJ/kg\right]$$

The specific enthalpy at the outlet of the expansion valve on the main refrigerant circuit (h_6_ [kJ/kg]) is equal with the specific enthalpy at the inlet:

(15) $$h_{6}=h_{5}\left[kJ/kg\right]$$

The parameters of the saturated vapors of the refrigerant at the outlet of the generator (state 2) were determined at the condensing pressure (p_2_ = p_k_) and temperature (t_2_ = t_k_).

The parameters of the saturated liquid weak solution at the outlet of the generator (state 3) were determined at the condensing pressure (p_3_ = p_k_) and at the temperature at the outlet of the generator (t_3_ = t_g_).

The parameters of the saturated liquid rich solution at the outlet of the absorber (state 9) were determined at the evaporating pressure (p_9_ = p_0_) and at the condensing temperature (t_9_ = t_k_).

The specific enthalpy of the liquid rich solution at the outlet of the pump (state 10) was determined as a function of the specific mechanical work of the pump (w_P_ [kJ/kg]) as:

(16) $$h_{10}=h_{9}+w_{P}\left[kJ/kg\right]$$

with

(17) $$w_{P}=\Delta p\cdot v_{9}\left[kJ/kg\right]$$

where (Δp = p_k_ – p_0_) and v_9_ [m^3^/kg] is the specific volume of the liquid rich solution at the inlet of the pump.

The parameters of the saturated liquid rich solution in the concentrating column of the generator (state L) were determined at the condensing pressure (p_L_= p_k_) and at the concentration of the rich solution.

The parameters of the saturated vapors in equilibrium with the rich solution in the concentrating column of the generator (state V) were determined at the condensing pressure (p_V_= p_k_) and at the equilibrium temperature of the rich solution and of the vapors (t_V_ = t_L_).

The temperature of the subcooled liquid rich solution at the inlet of the generator (t_1_ [ °C]) was determined as a function of the temperature of the saturated liquid (state L):

(18) $$t_{1}=t_{L}-3\left[{}^{\circ }C\right]$$

The recirculating factor (f [-]), representing the ratio between the flow rate of the rich solution (${\dot{m}}_{r}\left[kg/s\right]$) and the flow rate of the refrigerant in the main refrigerating circuit ($\dot{m}\left[kg/s\right]$), was determined as a function of the concentrations of NH_3_ in the main refrigerating circuit (ξ”=1), in the rich solution circuit (ξ_r_) and in the weak solution circuit (ξ_w_):

(19) $$f=\frac{\xi^{\prime \prime }-\xi_{w}}{\xi_{r}-\xi_{w}}=\frac{1-\xi_{w}}{\xi_{r}-\xi_{w}}$$

where (ξ_w_ = ξ_3_) and (ξ_r_ = ξ_9_).

The specific enthalpy of the subcooled weak solution at the inlet of the expansion valve (h_11_ [kJ/kg]), was determined from the energy balance equation on the heat exchanger on the NH_3—_H_2_O solutions circuit:

(20) $$h_{11}=h_{3}-\frac{f}{f-1}\cdot \left(h_{1}-h_{10}\right)\left[kJ/kg\right]$$

The specific enthalpy at the outlet of the expansion valve on the weak solution circuit (h_12_ [kJ/kg]) is equal with the specific enthalpy at the inlet:

(21) $$h_{12}=h_{11}\left[kJ/kg\right]$$

The specific enthalpy of the ideal pole of rectification (h_Pi_ [kJ/kg]) was determined as a function of the enthalpies (h_L_ [kJ/kg]) and (h_V_ [kJ/kg]) and of the concentrations of NH_3_ (ξ_L_ [kJ/kg]) and V (ξ_L_ [kJ/kg]) in the states L and V, respectively:

(22) $$h_{Pi}=\frac{h_{V}\cdot \left(1-\xi_{L}\right)-h_{L}\cdot \left(1-\xi_{V}\right)}{\xi_{V}-\xi_{L}}$$

The specific thermal power of the absorber (q_AB_ [kW/kg]) could be determined from the energy balance on the absorber:

(23) $$q_{Ab}=h_{8}+\left(f-1\right)\cdot h_{12}-f\cdot h_{9}$$

The specific thermal power of the ideal rectifier (q_Ri_ [kW/kg]) that should be evacuated from the rectifier (located in the upper side of the generator's concentrating column), could be computed as:

(24) $$q_{Ri}=h_{Pi}-h_{2}$$

The specific thermal power of the real rectifier (q_R_ [kW/kg]) was determined considering an efficiency of the rectification (η_r_ = 0.88):

(25) $$q_{R}=\frac{q_{Ri}}{\eta_{r}}$$

The specific thermal power of the generator (q_G_ [kW/kg]) could be determined from the energy balance on the generator:

(26) $$q_{G}=q_{R}+\left(f-1\right)\cdot h_{12}-f\cdot h_{9}$$

The specific thermal power of the condenser (q_k_ [kW/kg]) could be determined from the energy balance on the condenser:

(27) $$q_{k}=h_{2}-h_{4}$$

The specific thermal power of the evaporator (q_0_ [kW/kg]) could be determined from the energy balance on the evaporator:

(28) $$q_{0}=h_{7}-h_{6}$$

The coefficient of performance (COP [-]) could be determined as the ratio between the specific thermal powers of the evaporator and of the generator:

(29) $$COP=\frac{q_{0}}{q_{G}}$$

The thermal power of all the equipment were determined by multiplying the specific thermal powers with the mass flow rates corresponding to each equipment.

### Limits of the hot temperature at the outlet of the solar field

The working conditions of the absorption refrigeration plant are determined by the temperatures of the three heat sources:

- Inside temperatures of the cold food storage spaces (t_i_ [ °C])
- Temperature of the cooling water at the outlet of the cooling tower (t_w_ [ °C])
- Temperature of the thermal agent at the outlet of the SPTC (also named the hot temperature) (t_h_ [ °C]).

During the operation, it was considered that the inside temperatures of the cold food storage spaces were constant (3 °C for the RC and RS and −20 °C for the FC and FS).

Since the temperature of the cooling water is variable under the influence of the ambient conditions (t_db_ and φ), the hot temperature must be adjusted to maintain a minimum degassing zone (Δξ), representing the difference between the concentrations of the rich solution (ξ_r_) and of the weak solution (ξ_w_):

(30) $$\Delta \xi =\xi_{r}-\xi_{w}$$

A minimum degassing zone must be maintained because a decrease in degassing zone, will determine an increase in flow rates of the weak and rich solutions.

The hot temperature was determined for both refrigerating and freezing, considering two minimum values of the degassing zone (Δξ = 0.06) and (Δξ = 0.1).

**Fig. 6:**
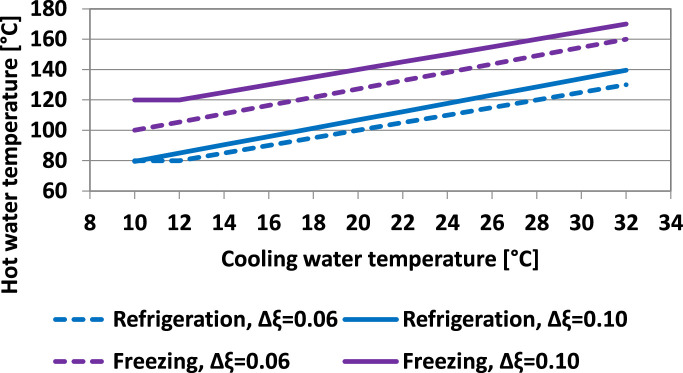
The minimum required hot temperatures as a function of the cooling water temperature, for refrigeration and freezing.

Fig. 6 presents the minimum required hot temperatures as a function of the cooling water temperature for the degassing zones (Δξ = 0.06) and (Δξ = 0.1), for refrigeration and freezing.

If the degassing zone is lower, for each value of the hot temperature, the range of compatible cooling water temperatures decreases.

Based on the presented investigation, the minimum required hot temperature (of the heat transfer fluid at the outlet of the SPTC) was determined as a function of the cooling water temperature (at the outlet of the cooling tower), for the two considered degassing zones and for both refrigeration and freezing operating regimes, using the relation:

(31) $$t_{h}=a\cdot t_{w}+b$$

with the values of the coefficients, a and b presented in Table 5.

**Table 5:** Values of the coefficients a and b (Eq. (31)).

Regime	Refrigeration (t_0_ = −10 °C)		Freezing (t_0_ = −30 °C)	
Δξ [-]	0.06	0.10	0.06	0.10
a	2.500	2.727	2.727	2.500
b	50.00	52.27	72.73	90.00
Range	t_w_ ≥ 12 °C	t_w_ ≥ 10 °C	t_w_ ≥ 10 °C	t_w_ ≥ 12 °C

To the best of the authors' knowledge, such a recommendation to calculate the minimum required hot temperature as a function of the cooling water temperature is not available in the literature.

The required hot temperatures are higher for higher degassing zones and for lower cooling temperatures.

### Coefficient of performance for the absorption refrigeration plant

The coefficient of performance (COP) of the absorption refrigeration plant, depends on the operating conditions represented by the temperatures of the three heat sources. In both refrigeration and freezing operating regimes the inside temperatures of the cold food storage spaces were considered constant (3 °C for the RC and RS and −20 °C for the FC and FS). Previously it was determined that the minimum required hot temperature depends on the cooling water temperature. Thus, it can be concluded that if the hot temperature is maintained at the minimum required value, COP depends only on the cooling water temperature, for each inside temperature.

**Fig. 7:**
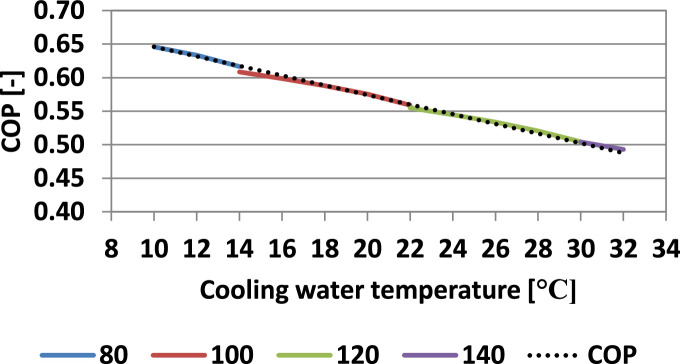
Influence of the cooling water temperature on the COP (refrigeration) (evaporating temperature: −10 °C).

**Fig. 8:**
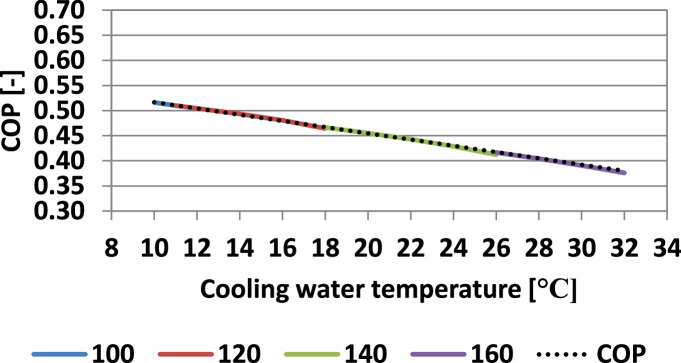
Influence of the cooling water temperature on the COP (freezing) (evaporating temperature: −30 °C).

Figs. 7 and 8 present the influence of the cooling water temperature on the COP considering the minimum required values of the hot temperatures for refrigeration and freezing.

In both refrigeration and freezing operating regimes, the COP dependence on the cooling water temperature is linear, and the following correlation was determined:

(32) $$COP=c\cdot t_{w}+d$$

with the values of the coefficients, c and d presented in Table 6.

**Table 6:** Values of the coefficients c and d (Eq. (32)).

Regime	c	d
Refrigeration (t_0_ = −10 °C)	−0.0072	0.7182
Freezing (t_0_ = −30 °C)	−0.0062	0.5786

To the best of the authors' knowledge, such a recommendation to calculate the COP as a function of the cooling water temperature, for the minimum hot temperature to maintain a reasonable degassing zone, is not available in the literature.

### Coefficient of performance for the electric refrigeration plant

The coefficient of performance (COP) of the electric refrigeration plant, representing the ratio between the cooling power and the electrical power consumed for compression, depends on the operating conditions represented by the temperatures of the two heat sources (the inside temperature of the cold food storage spaces and the cooling water temperature). Like in the case of the absorption refrigeration plant, in both refrigeration and freezing operating regimes, the inside temperatures of the cold food storage spaces were considered constant (3 °C for the RC and RS and −20 °C for the FC and FS). Under these circumstances it can be concluded that the COP depends only on the cooling water temperature, for each inside temperature. COP was calculated for a classical mechanical compression cycle with NH_3_ as refrigerant.

**Fig. 9:**
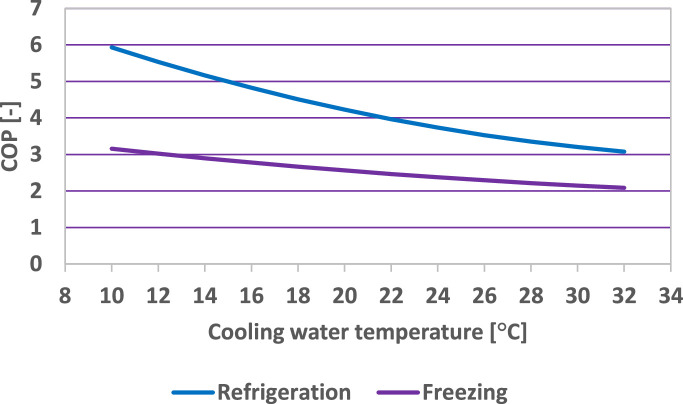
COP variation with the cooling water temperature for refrigeration and freezing.

Fig. 9 presents the COP variation with the cooling water temperature for refrigeration and freezing operating regimes, respectively.

The values of the COP are higher when the refrigeration plant operates in the refrigeration regime in comparison to the freezing regime.

The correlation between the COP and the cooling water temperature, for NH_3_ as refrigerant was determined as:

(33) $$COP=e\cdot t_{w}^{2}+f\cdot t_{w}+g$$

with the values of the coefficients e, f and g presented in Table 7.

**Table 7:** Values of the coefficients e, f and g (eq. (33)).

Regime	e	f	g
Refrigeration (t_0_ = −10 °C)	0.0034	−0.2724	8.3136
Freezing (t_0_ = −30 °C)	0.0009	−0.0863	3.9280

The presented correlation with the calculated coefficients was used to determine the COP of the backup mechanical refrigerating plant, needed to be used when solar heat is not available or is insufficient.

The mathematical model characterizes the thermal behaviour of all the investigated solar cooling system components: the cold food storage spaces (RC, RS, FC, FS), the food product itself (chicken meat), the parabolic trough collectors, the cooling tower, the main absorption refrigeration system and the backup mechanical refrigeration system.

The different parts of the proposed mathematical model were implemented in Engineering Equation Solver (EES) and in Microsoft Excel.

## Discussion and conclusions

The study presents the methodology for the analytical investigation of the food storage cooling with solar ammonia-water absorption system, powered by SPTC. To the best of the authors' knowledge, the study is investigating the following novel aspects:

- Food cooling with solar absorption refrigeration system.
- Hourly based modelling of food cooling load variation.
- Hourly based variation of NH_3—_H_2_O solar absorption system performances.
- Long term simulation of solar absorption cooling for refrigeration and cooling.

The thermal behaviour of four types of food storages: RC, RS, FC and FS and of the heat storage tank was investigated.

The results of the methodology application are going to be presented in a distinct study.

## Declaration of Competing Interest

The authors declare that they have no known competing financial interests or personal relationships that could have appeared to influence the work reported in this paper.

## Data Availability

Data will be made available on request.
